# Reduction of the concentration of particulate material at a sampling point in Cusco city at the beginning of the pandemic

**DOI:** 10.1038/s41598-023-50955-y

**Published:** 2024-01-08

**Authors:** Julio Warthon, Modesta Alvarez, Amanda Olarte, Yanett Quispe, Victor Jalixto, Nazaria Valencia, Mirian Mio-Diaz, Ariatna Zamalloa, Bruce Warthon

**Affiliations:** 1 Departamento Académico de Física, Universidad Nacional de San Antonio Abad del Cusco, Av. De La Cultura 733, Cusco, 08003 Peru; 2 Departamento Académico de Biología, Universidad Nacional de San Antonio Abad del Cusco, Av. De La Cultura 733, Cusco, 08003 Peru; 3 Departamento Académico de Química, Universidad Nacional de San Antonio Abad del Cusco, Av. De La Cultura 733, Cusco, 08003 Peru

**Keywords:** Environmental impact, Public health, Atmospheric science

## Abstract

The pandemic produced by SARS-CoV-2 generated various impacts on public health, the environment and other anthropogenic activities. The purpose of this study was to evaluate the reduction of air pollution due to $PM_{2.5}$ and $PM_{10}$ particulate matter in Cusco city at the beginning of the pandemic. Social confinement in Peru began on March 16, 2020, until the end of June. These health measures caused strict isolation that resulted in a significant decrease in vehicle flow on the streets and avenues of the city of Cusco. In the first days of May, even during the time of confinement, we managed to measure air quality at a sampling point located on the campus of the Universidad Nacional de San Antonio Abad de Cusco; a reduction in air pollution due to particulate matter was observed. The evaluation was carried out using an high-volume (HiVol) 3000 particulate matter sampler and the mass of particulate matter adhered to the filters was determined by gravimetry. The concentrations of particulate matter $PM_{2.5}$ and $PM_{10}$ obtained pre-pandemic were compared with those recorded at the beginning of the pandemic. The results revealed a significant average reduction in the concentration of $PM_{10}$ and $PM_{2.5}$, reaching − 57.43% and − 59.52%, respectively, compared to pre-pandemic values. At the same time, its relationship with meteorological parameters and Google mobility data was evaluated and it was concluded that these parameters did not significantly affect the reduction of particulate matter. This study reveals the positive effects of the pandemic in reducing air pollution and the confinement measures had as a secondary effect on the decrease in air pollution in Cusco City.

## Introduction

The outbreak of the pandemic in late 2019 with the introduction of the new coronavirus, SARS-CoV-2^1^, sparked a series of notable events. On March 11, 2020, the World Health Organization (WHO) declared the COVID-19 disease a global health emergency, leading to global restrictions aimed at mitigating the spread of the virus^2,3^. This epidemiological context induced a significant reduction in anthropogenic emissions, especially in the transportation and industry sectors^4–7^. The confinements and measures taken during the pandemic exhibited beneficial effects on air quality. Research-based on satellite data revealed reductions in both concentrations and emissions of air pollutants globally^8–11^. Through satellite studies, decreases in air pollution were identified in geographic regions such as Europe, Asia and America, specifically in the months of March and April 2020^12,13^. Air pollution represents a major environmental risk to human health, causing millions of premature deaths each year due to exposure to fine particulate matter, as reported by the World Health Organization^14^. This problem has increased in recent decades mainly due to anthropogenic activity, especially combustion in motor vehicles, which generates chemicals such as greenhouse gases and particulate matter^15–17^. According to Baird and Cann^18^, the particulate material present in the atmosphere has a natural or anthropogenic origin, the diameter is between the range of 2 nm and 100 μm. Some of these particles are toxic, producing harmful effects for humans who inhale them^16^. According to the WHO guidelines (2006) for fine particles ($PM_{2.5}$) is 25 μg/m^3^ average in 24 h, and for coarse particles ($PM_{10}$) 50 μg/m^3^ average in 24 h^19^, these criteria were in force at the beginning of the pandemic until 2021, the current WHO guidelines (2021) for fine particles ($PM_{2.5}$) is 15 μg/m^3^ 24-h average, for coarse particles ($PM_{10}$) 45 μg/m^3^ 24-h average^20^. The confinement caused a marked reduction in mobility, especially in the automotive transport sector, the latter being notably affected by the restrictions implemented around the world^21,22^. Various studies have demonstrated the effects of social confinement on atmospheric quality. A survey carried out in Volos, Greece, has shown a substantial reduction of 37.4% in the $PM_{2.5}$ pollution load throughout the three phases of the COVID-19 pandemic in 2020, compared to 2019^23^. Another study carried out in Western Macedonia, Greece, noted that the concentrations of $PM_{2.5}$ and $PM_{10}$ experienced a predominant decrease in the years 2020 and 2021 during the lockdown periods, a decrease was found in the average concentrations of $PM_{2.5}$ that varied between − 1 and − 36%, while the average concentrations of $PM_{10}$ showed an even more pronounced reduction, fluctuating between − 13 and − 40%^24^. A study carried out in Bhubaneswar, India, throughout the entire lockdown phase, revealed a 42.35% reduction in $PM_{10}$ and 45% in $PM_{2.5}$^25^. In Peru, the authorities implemented mandatory confinement on March 16, 2020, as a measure to contain the spread of COVID-19^26^. In cities such as Arequipa and Lima, exhaustive studies were carried out to evaluate air quality during the pandemic period. In the metropolitan area of Lima, particles ($PM_{2.5}$ and $PM_{10}$), NO2, and O3 were analyzed, where significant decreases were observed in the concentrations of $PM_{10}$ (− 40% and − 58%) and $PM_{2.5}$ (− 31% and − 43%)^27^. In the case of Arequipa, the concentrations of $PM_{2.5}$ and $PM_{10}$ were compared before and during the pandemic, showing a notable decrease in the concentration of $PM_{2.5}$ by 21.0% and $PM_{10}$ by 21.5%^28^. Cusco is a city in southern Peru that has climatic and geographical particularities different from the cities previously studied, such as its high altitude and unique geography. The research carried out during the pandemic constitutes a contribution to the studies carried out in the region and the global context. In Cusco City, greenhouse gas (GHG) and particulate matter (PM) emissions come mainly from automotive vehicles and kilns for brick production^29,30^. In recent years, air pollution in the city has increased proportionally with the increase in vehicular transportation and brick kilns^29,31,32^. Furthermore, ambient air quality is intrinsically linked to various factors, including emissions from vehicular and industrial activities, local meteorological conditions, and natural processes^33^. Regarding the correlation of meteorology with PM, uncertainty persists in various contexts, given the diversity of components of said particles^34^. In Cusco city, the climate is characterized by two typical seasons, a rainy season and a dry season. The information available on pollution by particulate matter in Cusco city is scarce. It is relevant to highlight that the Universidad Nacional de San Antonio Abad del Cusco (UNSAAC) is the only institution that during the pandemic had the HiVol 3000 particulate matter sampler with an interchangeable head to measure $PM_{2.5}$ and $PM_{10}$, which has allowed obtaining new and unique data. This research focused on evaluating the concentration of suspended particles, specifically $PM_{2.5}$ and $PM_{10}$, in two time periods: before and during the restrictions derived from the COVID-19 pandemic in Cusco. The main purpose was to carry out an analysis of the effects induced by confinement measures, which resulted in an appreciable decrease in mobility and human activities, impacting air quality at ground level. The results obtained represent a relevant contribution to knowledge in the field of atmospheric pollution, in addition to offering an understanding of the indirect effects of the pandemic, they allow us to investigate the influence of meteorological parameters and human activity, specifically the impact of the vehicle fleet, on concentrations of particulate matter. It was observed that anthropogenic activity directly influenced atmospheric pollution; on the other hand, the quantitative results will contribute to a better understanding of air pollution processes in times of pandemic.

## Methodology

### Particulate matter sampling point

**Figure 1:**
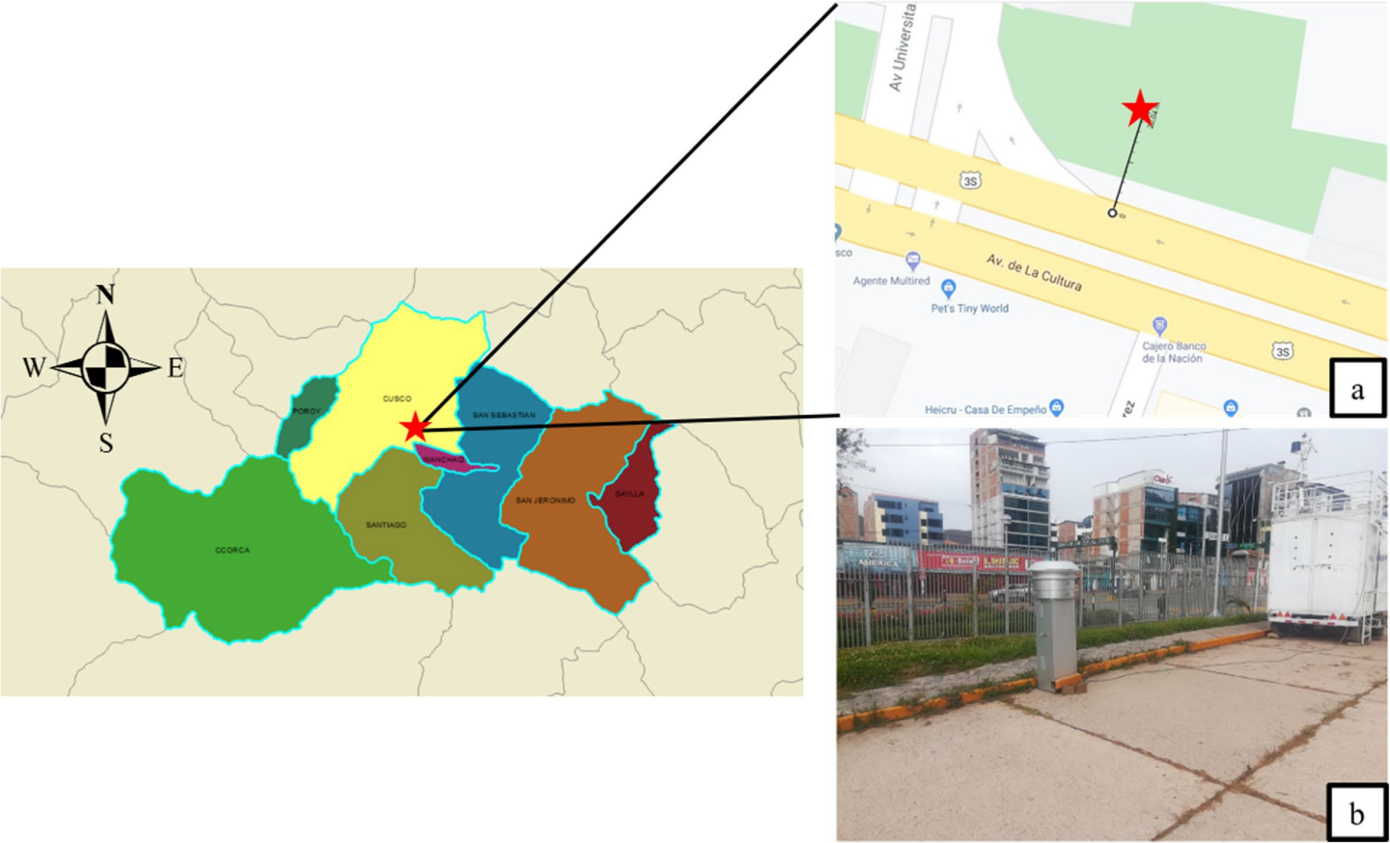
Sample point located on the Universidad Nacional de San Antonio Abad del Cusco campus (Map was created using Esri ArcMap 10.8.0.12790; http://esri.com).

This study was carried out in Cusco city, which is located in the southern region of Peru, surrounded by mountains and located at an average altitude of 3350 meters above sea level^35,36^. The sampling site was established on the campus of the Universidad Nacional de San Antonio Abad del Cusco, adjacent to Avenida de la Cultura considered a high vehicular traffic route (Fig. 1a). The precise geographical coordinates of the sampling point are Latitude − 13.5225 and Longitude − 71.96083. This monitoring point meets the requirements stipulated in the Code of Federal Regulations of the United States Environmental Protection Agency (CFR 40), which establishes the representativeness criteria for the location of sampling sites for total suspended particles. The particle sampling equipment was installed at a distance of 20 m from the edge of the road on Avenida de la Cultura; there were no obstacles around the equipment that could influence the sampling.

### Particulate matter data

A high-volume particulate matter sampler with an HiVol 3000 interchangeable head was used to measure $PM_{2.5}$ and $PM_{10}$ from the ECOTECH brand^37^.

The protocols established by the EPA^38^ and the Ministerio del Medio Ambiente del Peru (MINAM, 2019)^39^ were followed. The amount of particulate matter deposited in the quartz filters was determined using gravimetry^38,40,41^. The filters were placed in a drying hood for 24 h. Next, they were weighed on an OHAUS brand analytical balance Pioneer PA224 with a sensitivity of 0.0001 g. Four measurements of the initial mass ($m_i$) of each filter were made to obtain a reliable average. After these weighings, the filter was immediately placed in an airtight container and transported from the laboratory to the sampling site, located about 120 m away. The airflow recorded by the machine, pressure, and ambient temperature were verified. Simultaneously, the start time of the test was recorded.

After 24 h of sampling, the filter with the deposited particulate material was removed and taken to the laboratory to be placed in the drying hood for one day. Finally, the final mass ($m_f$) of the filter containing particulate matter was measured. The mass difference $m_f - m_i = m$ represents the mass of the particulate material obtained during sampling. The concentration of particulate matter was evaluated by the ratio of the mass of the pollutant (*m*) between the standard volume $V_{std}$^42^, using the equation:

1 $$\begin{aligned} C = \frac{m}{V_{\text {std}}} \end{aligned}$$

The standard volume was determined using the equation established in method IO-2.4^42^:

2 $$\begin{aligned} V_{\text {std}} = V_s \cdot \frac{P_{\text {atm}}}{P_{\text {std}}} \cdot \frac{T_{\text {std}}}{T_{\text {atm}}} \end{aligned}$$

The temperature $T_{atm}$ corresponds to the sampling location, in this research it corresponds to Cusco city where it was measured with Hg thermometers and digital thermometers, and the atmospheric pressure $p_{atm}$ was measured with a flow meter (flow calibrator, Mesa Labs brand, FlexCal model), the temperature $T_{std}$ and standard pressure p std are constant values established in the EPA protocol (1999)^42^: $T_{std}$ = 298 K and $p_{std}$ = 760 mmHg. Equation (2) was applied at the sampling point under pressure and temperature conditions other than STP (Standard temperature and pressure)^43^. The volume of uncorrected accumulated air sample in Cusco city at an altitude of 3347 meters above sea level was obtained using the equation:

3 $$\begin{aligned} V_s = Q \cdot t \end{aligned}$$

The flow rate *Q* is measured instantaneously by the HiVol 3000 sampler. The flow of atmospheric air through the sampling equipment was 67 $m^3/h$, the flow was approximately constant for 24 continuous hours. The daily values and their averages were obtained in this way.

### Study period

Measurements before the start of the pandemic were carried out in January and February 2020. During the months of March and April, it was not possible to carry out measurements due to the total confinement generated by the COVID-19 pandemic. Sampling resumed on May 2, 2020, 6 weeks after the start of the pandemic, amid conditions of social isolation, until July 27, 2020.

**Figure 2:**
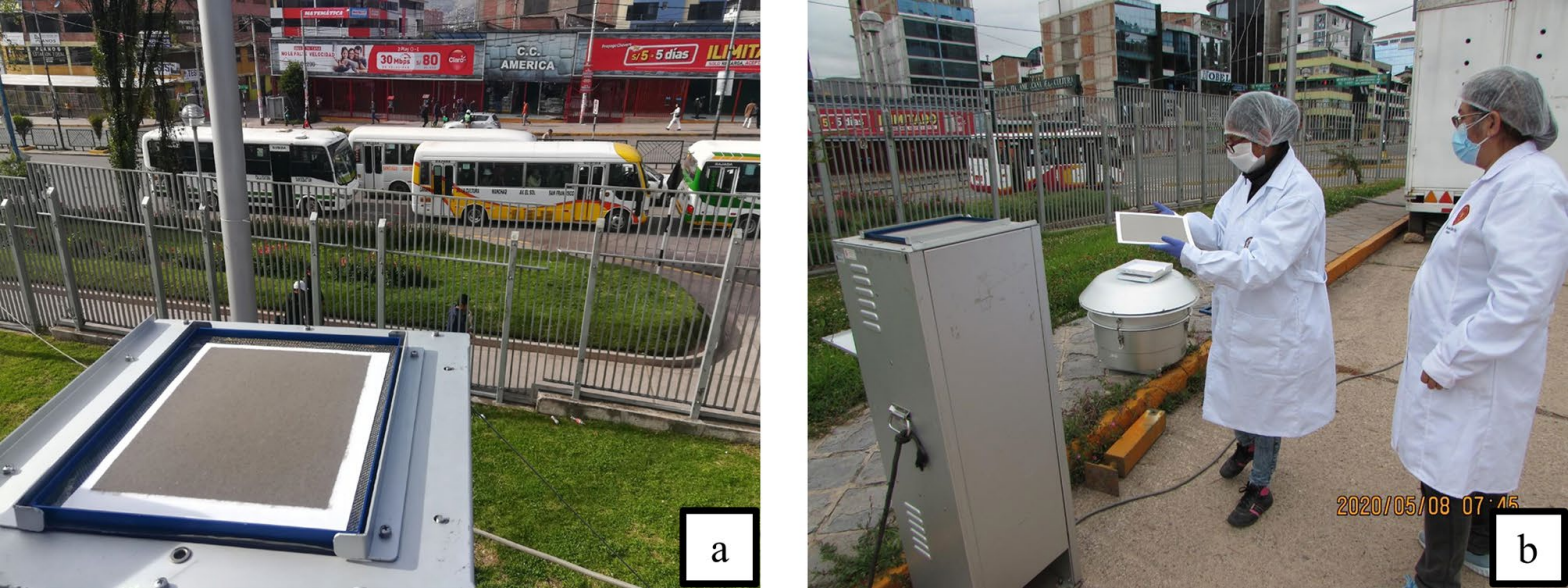
Particulate matter filters.

Figure 2a represents automotive transportation in regular conditions before the pandemic, specifically on Monday, January 27, 2020. In contrast, Fig. 2b portrays the scenario on Friday, May 8, 2020., during the confinement period, evidencing a significant reduction in motor transport activity compared to the period before the pandemic.

### Mobility data

The mobility data used in this study comes from local mobility reports provided by Google^44^, collected from February 15, 2020, to July 27, 2020, coinciding with the end of the data collected from the defined study period. These data focus on the evaluation of mobility behavior in the Cusco region during a critical period, from weeks before the start of the SAR-CoV2 pandemic.

### Meteorological parameters

The meteorological parameters considered in the study were the maximum and minimum temperatures obtained from the Senamhi–Kayra station^45^, for all evaluations carried out before the start of the pandemic. However, due to the restrictions imposed by the pandemic, some sectors, including the Kayra monitoring station, stopped storing data continuously. Due to this problem, it was decided to use datasets from the Earth Engine Data Catalog^46^ to Calculate the average air temperature 2 m above the ground. Additionally, maximum temperature, minimum temperature, and relative humidity data provided by the Alejandro Velasco Astete International Airport station were integrated, through the OGIMET platform^47^ and the GHCN-Daily NOAA satellite^48^.

## Results and discussion

### Concentration of $PM_{2.5}$ and $PM_{10}$ in 2020

Table 1 shows the concentrations of particulate matter $PM_{2.5}$ and $PM_{10}$ measured in the period before the pandemic, the values during the months of January and February—rainy season in Cusco city^49^—reached levels above the air quality standards (ECA) for $PM_{2.5}$, in January the average was 58.21 μg/m^3^, in the case of $PM_{10}$ it was 45.86 μg/m^3^ lower than the ECA. In the month of February, the average concentration of $PM_{10}$ was 56.47 μg/m^3^, a value higher than the WHO RCTs of 2006 and 2021.

**Table 1:** $PM_{2.5}$ and $PM_{10}$ concentration data (pre-pandemic).

Particulate matter 2.5			Particulate matter 10		
Date	Season	Concentration ($\mu$g/m^3^)	Date	Season	Concentration ($\mu$g/m^3^)
25-01-2020		69.39 ± 15.73	29-01-2020		56.28 ± 10.57
27-01-2020	Rainy	47.38 ± 7.50	30-01-2020		39.53 ± 6.50
28-01-2020	Season	57.87 ± 9.23	31-01-2020	Rainy	41.24 ± 7.38
			13-02-2020	Season	36.67 ± 17.56
			14-02-2020		71.89 ± 12.23
			17-02-2020		43.77 ± 14.32
			18-02-2020		73.54 ± 19.12

Table 2 shows the concentration of $PM_{2.5}$ and $PM_{10}$ evaluated in the months of May, June, and July, which corresponds to the dry season in Cusco city^49^. The average concentration value of $PM_{10}$ in the first week from May 3 to 5 was 22.07 μg/m^3^, the average concentration of $PM_{2.5}$ from May 6 to 9 was 18.87 μg/m^3^, it is observed that the concentration values in both cases were below the usual measures taken before the pandemic.

**Table 2:** $PM_{2.5}$ and $PM_{10}$ Concentration data during the first months of the pandemic.

Particulate matter 2.5			Particulate matter 10		
Date	Season	Concentration ($\mu$g/m^3^)	Date	Season	Concentration ($\mu$g/m^3^)
07-05-2020		20.85 ± 3.49	03-05-2020		8.18 ± 2.42
08-05-2020		20.66 ± 3.51	04-05-2020		23.95 ± 4.63
09-05-2020		18.66 ± 3.26	05-05-2020		35.54 ± 6.47
10-05-2020		15.61 ± 2.77	06-05-2020		20.61 ± 3.45
11-05-2020		18.59 ± 3.14	28-06-2020		40.31 ± 7.29
02-06-2020	Dry	54.32 ± 10.05	29-06-2020	Dry	60.58 ± 10.13
03-06-2020	Season	38.50 ± 7.66	30-06-2020	Season	64.33 ± 11.47
04-06-2020		46.53 ± 8.71	01-07-2020		66.17 ± 11.72
24-06-2020		56.45 ± 10.26	25-07-2020		43.66 ± 8.14
25-06-2020		73.91 ± 12.75	26-07-2020		42.96 ± 8.07
26-06-2020		57.84 ± 10.54	27-07-2020		79.56 ± 14.83
27-06-2020		53.34 ± 9.87			

### Variation of $PM_{10}$

**Figure 3:**
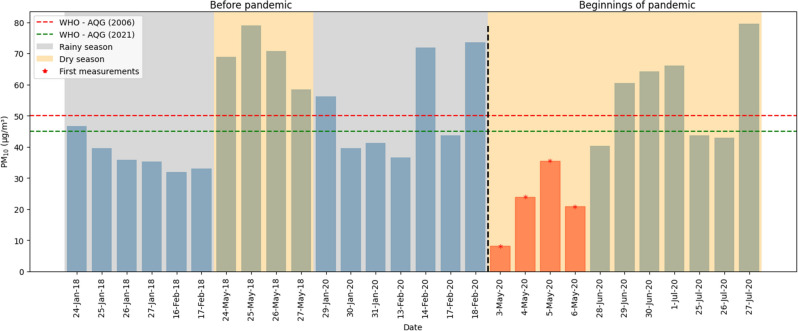
$PM_{10}$ before and at the beginning of the pandemic.

Figure 3 shows the variation of $PM_{10}$ concentration in 2018 and 2020 in the same periods, similar rainy and dry seasons. Before the pandemic, the average value of the $PM_{10}$ concentration in January was 45.68 μg/m^3^ and in February it was 56.47 μg/m^3^. The values during 2020 show that the maximum value obtained in this period was 73.54 μg/m^3^, a value above the WHO guidelines (2006)^19^ and current guidelines (WHO, 2021)^20^, on the other hand, the minimum value was 36.67 μg/m^3^. In the first days of May 2020, measurements of the $PM_{10}$ concentration were resumed, the average value was 22.07 μg/m^3^ between May 3 and 6, the maximum value was 35.54 μg/m^3^ and the minimum value was 8.18 μg/m^3^, all values are below the WHO^19^ and WHO^20^ guidelines.

The percentage difference between the maximum values was − 51.67%, which indicates a significant reduction in the concentration of $PM_{10}$ during the pandemic compared to the months of January and February 2020, before the pandemic. The percentage difference between the minimum values was − 77.69%, which demonstrates an even greater decrease in $PM_{10}$. Regarding the average value, a percentage reduction of − 57.43% was found. However, when comparing the concentration levels of particulate matter between 2018 and 2020 during the dry season in Cusco, an even more notable reduction in the average $PM_{10}$ concentration of 68.01% is observed. These data reveal a consistent trend in the decrease in particulate matter during the dry season of 2020, especially during the month of May, which stood out for presenting the most significant reductions in this concentration, thus marking an evident pattern of change in the air quality. The graph clearly shows this decrease in the concentration of particulate matter. It is observed that the measurements recorded during the dry season of 2018 exhibit considerably higher levels compared to the rainy season that developed during the months of January and February. This difference reflects a notable reduction in air quality during the dry season of 2020 in contrast to previous evaluations.

The progressive return of anthropogenic activities and automotive transportation occurred starting in May, as shown in Fig. 5. The increase in vehicular flow continued in June and July in Cusco city^50^, the Concentrations were increasing and returning to their pre-pandemic values. The average value in June was 57.85 μg/m^3^, and in July it was 55.39 μg/m^3^, these values demonstrate the notable increase compared to the values obtained at the beginning of the month of May, which was 22.07 μg/m^3^.

### Variation of $PM_{2.5}$

**Figure 4:**
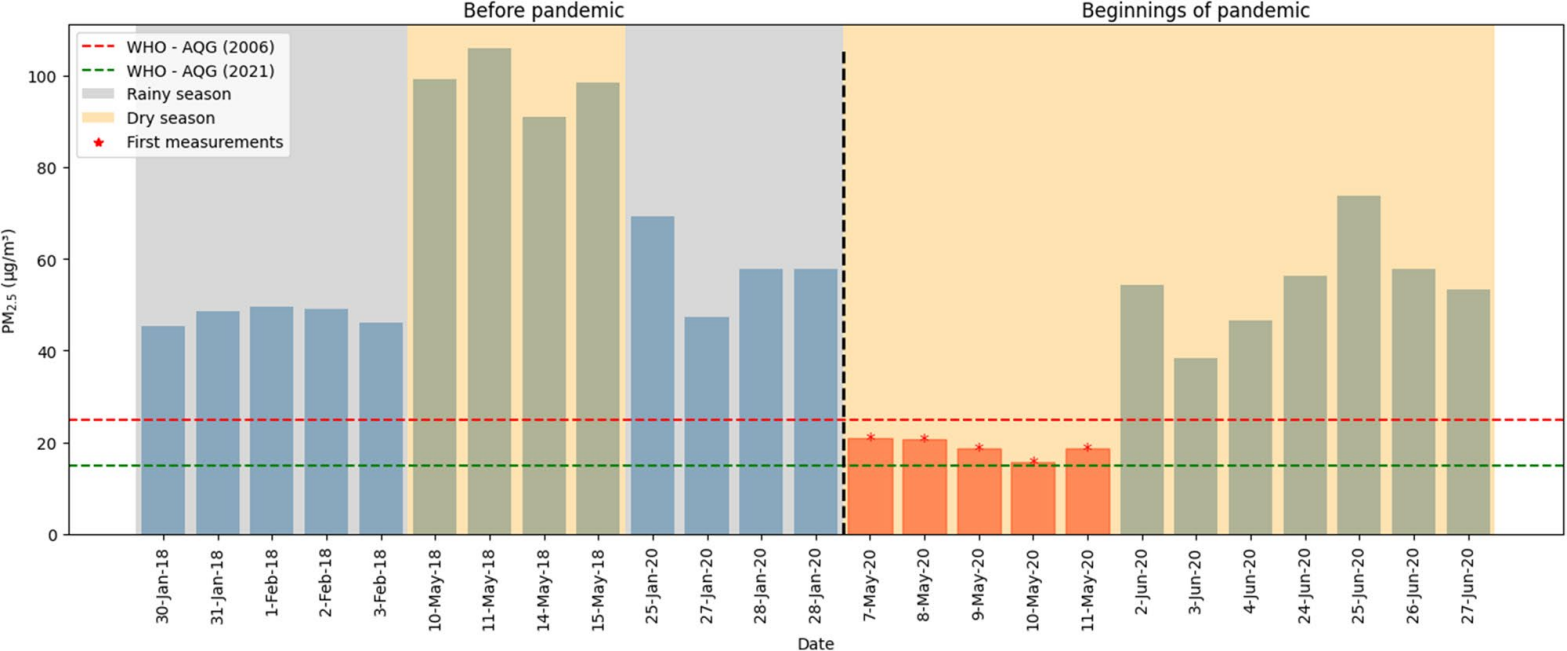
$PM_{2.5}$ before and at the beginning of the pandemic.

Figure 4 shows the fluctuations in $PM_{2.5}$ concentration in the years 2018 and 2020 at the same time. The concentration of particulate matter months before the start of the pandemic shows values that are above the WHO guidelines, even when it is the rainy season. During total confinement, both nationally and globally^51^, concentrations could not be measured due to restrictions inherent to the health emergency. At the beginning of May, measurements were resumed, which coincided with the final stage of confinement; concentration values were obtained that were below the WHO guidelines.

Figure 3 shows the variation of $PM_{10}$ concentration in 2018 and 2020 in the same periods, similar rainy and dry seasons. Before the pandemic, the average value of the $PM_{10}$ concentration in January was 45.68 μg/m^3^ and in February it was 56.47 μg/m^3^. The values during 2020 show that the maximum value obtained in this period was 73.54 μg/m^3^, a value above the WHO guidelines (2006)^19^ and current guidelines (WHO, 2021)^20^, on the other hand, the minimum value was 36.67 μg/m^3^. In the first days of May 2020, measurements of the $PM_{10}$ concentration were resumed, the average value was 22.07 μg/m^3^ between May 3 and 6, the maximum value was 35.54 μg/m^3^ and the minimum value was 8.18 μg/m^3^, all values are below the WHO^19^ and WHO^20^ guidelines.

According to the graphic representation in Fig. 4, a comparative analysis was carried out with the values before the pandemic, corresponding to the month of January. In this period, it was observed that the maximum concentration value of $PM_{2.5}$ was 69.39 μg/m^3^, the minimum value was 47.38 μg/m^3^, and the average was 58.21 μg/m^3^. These values demonstrate a significantly high concentration even before the pandemic, exceeding the guidelines established by the World Health Organization (WHO, 2006)^19^ (guidelines in force at the beginning of the pandemic) and 2021^20^ (current guidelines).

In May 2020 during strict confinement, a significant decrease in the concentration of $PM_{2.5}$ was observed, the maximum concentration value recorded in May was 33.22 μg/m^3^, the minimum value was 15.61 μg/m^3^, and the average was 23.57 μg/m^3^; The results, shown graphically in Fig. 4, indicate that the minimum value is slightly above the current WHO guidelines at 0.61 μg/m^3^. However, in general terms, the concentration levels were below the guidelines set by the WHO in 2006.

The analysis of the data in Fig. 4 reveals a percentage reduction of − 52.13% between the maximum values of $PM_{2.5}$ between the pre-pandemic period and the May 2020 measurements, a percentage reduction of − 67% is observed in the minimum values of $PM_{2.5}$ during that same period. Regarding the average value, a percentage reduction of − 59.52% was found between the values obtained before the pandemic of the same year and those registered during the month of May 2020.

When comparing the concentration levels of $PM_{2.5}$ particulate matter during the dry season between 2018 and 2020, a reduction of 80.87% was evident during the confinement period associated with the pandemic. When analyzing the data between the dry and rainy seasons of 2018, it is observed that the highest levels were recorded during the dry season of that year. However, for the year 2020, it is found that, in the same dry season, significant reductions in the levels of $PM_{2.5}$ particulate matter have been experienced.

Subsequently, with the progressive return of vehicles in Cusco city^50^, it can be seen (Fig. 4) that the $PM_{2.5}$ values returned to normal in the months of June and July. The percentage difference between the maximum values before the pandemic of the same year 2020 and during the first months of the pandemic was + 6.51%. The percentage difference between the minimum values during that entire period was -67.05% and the average decreased percentage by − 34.79%.

### Mobility variation in Cusco

**Figure 5:**
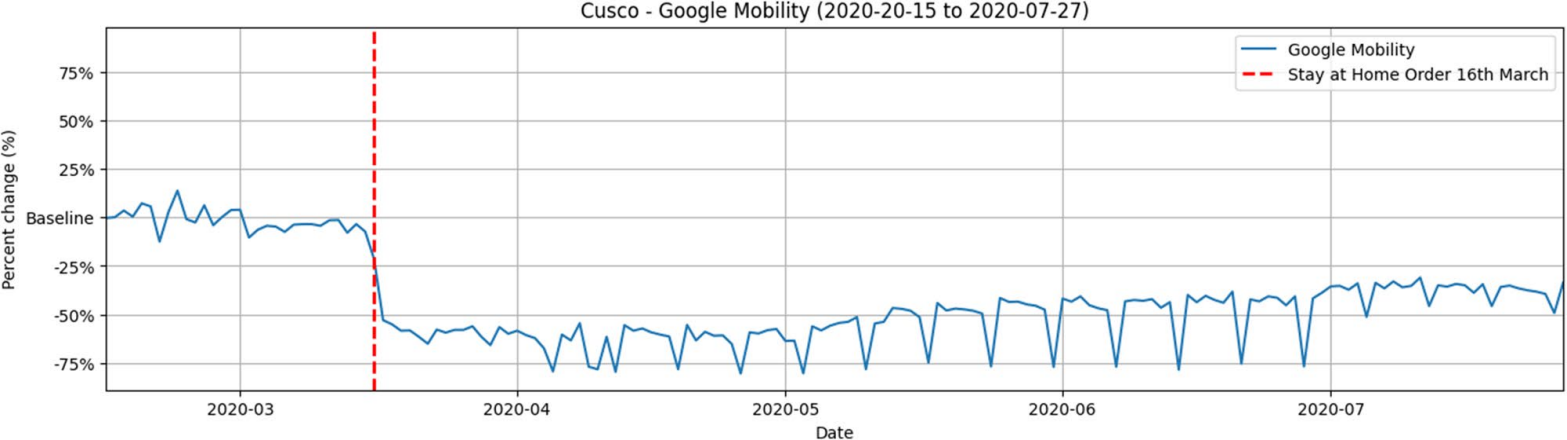
Mobility variation.

Figure 5 shows that as of March 16, when the confinement began, there was a decrease in the mobility of automotive transport in the city of Cusco. Since that turning point, stability was maintained in local mobility levels during the months of March and April, indicating that the population responded to the call to stay in their homes, which resulted in a notable reduction in mobility in the city. However, as we move towards the months of June and July, a change in this trend is beginning to be noticed. During this period, reports reflect a gradual increase in local mobility levels.

### Evaluation of $PM_{2.5}$ and $PM_{10}$ with respect to meteorological variables

**Figure 6:**
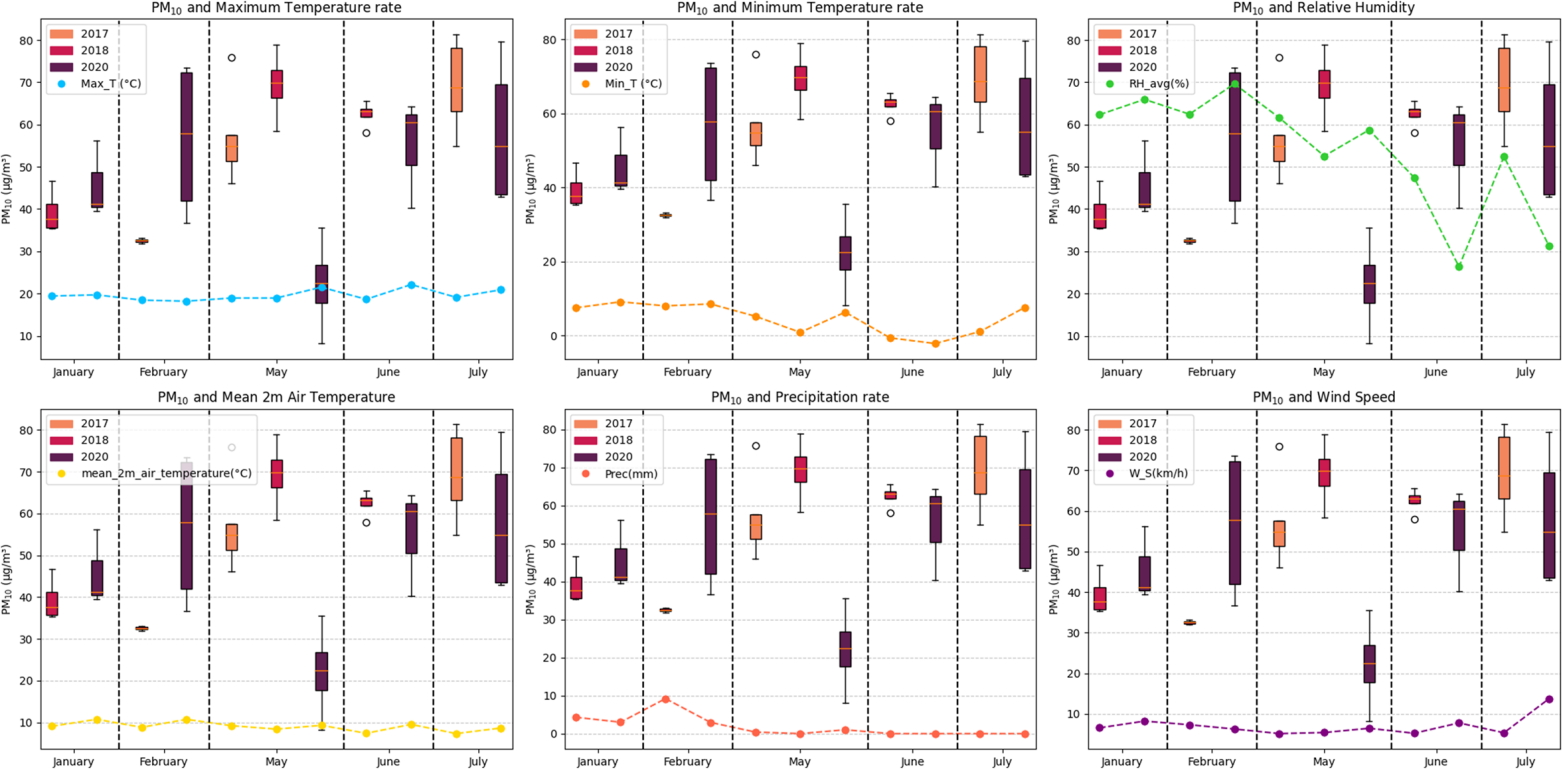
Particulate matter and meteorological variables.

Figure 6 describes an analysis of the meteorological variables and the average $PM_{10}$ concentrations before the pandemic and those obtained in the study corresponding to the beginning of the pandemic. Measurements from previous years at the same site and at locations close to the sampling point were compared. The figure highlights the drastic change in the concentration of particulate matter during the month of May, that is, during the first measurements of $PM_{10}$ and later for the months of June and July the values tend to typical quantities, this aspect was observed in the Fig. 3 It was observed that the months of January and February the meteorological variables showed changes typical of their non-linear nature, the concentrations of $PM_{10}$ were higher in 2020 compared to 2018. Graphically, it is evident that this change is not strongly linked to meteorological variables, but rather to the decrease in vehicular traffic seen in Fig. 2 and the mobility of people (Fig. 5).

**Figure 7:**
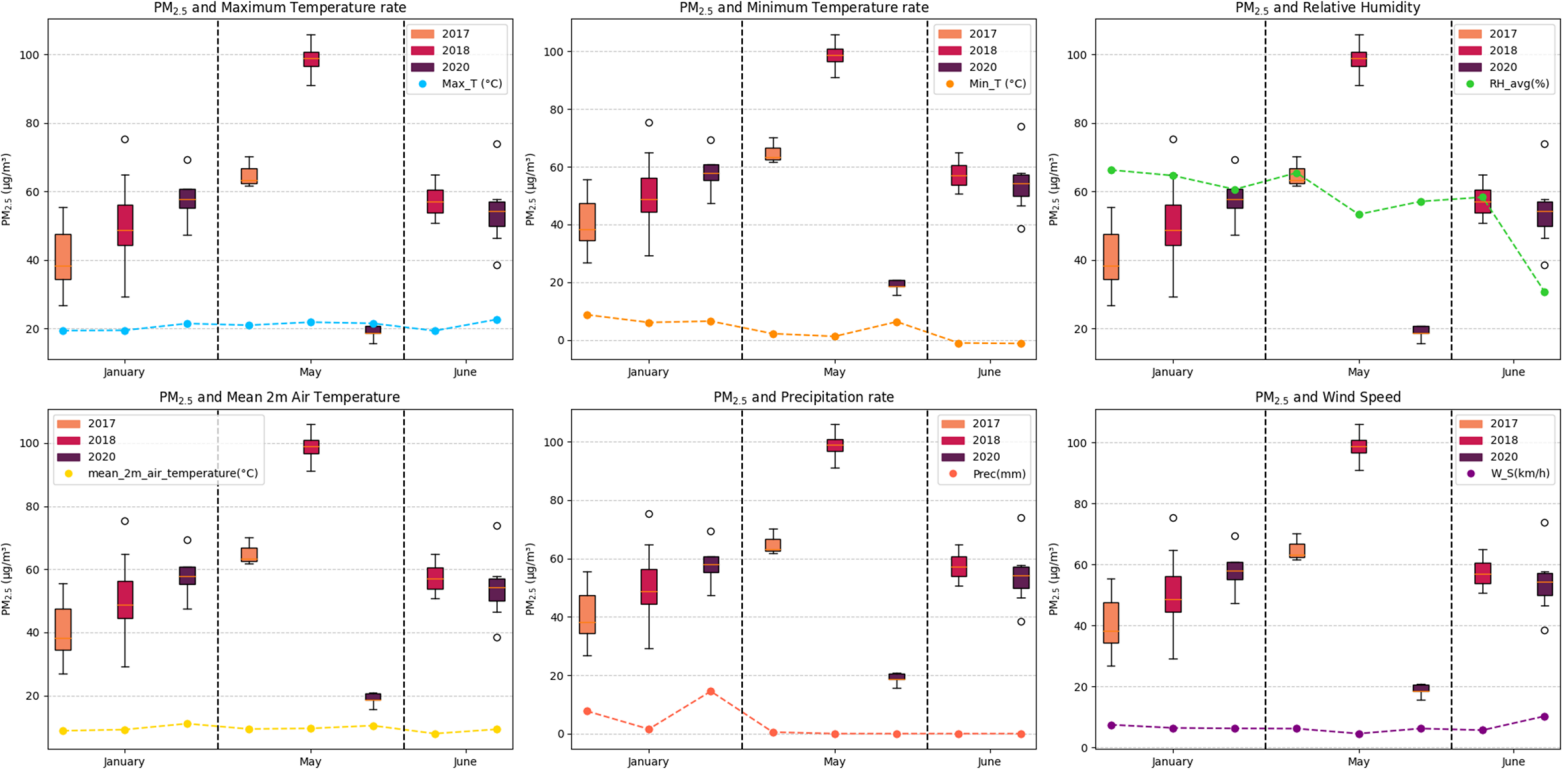
Particulate matter and meteorological variables.

Figure 7 shows a comparison between the average $PM_{2.5}$ concentrations before the pandemic and those used in the study during the pandemic. Like $PM_{10}$, the graph highlights a significant change in the concentration of particulate matter during the month of May, declining drastically due to the confinement measures implemented. Graphically, it is observed that this change is not directly related to meteorological variables, unlike other months.

Table 3 presents the results of the Pearson correlation between the meteorological parameters and the concentrations of $PM_{2.5}$ and $PM_{10}$. This correlation was based on daily particulate matter data relative to average daily weather data. Both cases present statistically significant correlations, with a p-value of less than 0.05. However, it is observed that the concentration of $PM_{2.5}$ shows a moderate and negative correlation with the minimum temperature (°C), relative humidity (%), and precipitation (mm/day). Furthermore, it presents a moderately positive correlation with the maximum temperature (°C). This pattern is not consistently replicated in the case of $PM_{10}$.

**Table 3:** Correlation analysis between meteorological parameters and $PM_{2.5}$/$PM_{10}$.

Meteorological parameters	$PM_{2.5}$		$PM_{10}$	
	Pearson correlation	P-value	Pearson correlation	P-Value
Max. temperature ($^\circ$C)	0.402219	0.013592	0.240522	0.040389
Min. temperature ($^\circ$C)	− 0.425215	0.008702	− 0.348719	0.002498
Relative humidity (%)	− 0.470381	0.003791	− 0.265365	0.023269
Precipitation (mm/day)	− 0.402653	0.013482	− 0.284304	0.014781

These results suggest that, although the concentration of particulate matter is influenced by meteorological variables, during the pandemic period there was no direct relationship, as illustrated in Figs. 6 and 7.

In contrast to other research such as that carried out in Colombia by Arregocés^52^, in which meteorological variables, such as the presence of precipitation, positively influenced the reduction of $PM_{2.5}$, in Risaralda and Bogotá, our results do not show a strong influence on the part of the meteorological parameters for the reduction of PM. Also taking into account that, during the dates of May 2020, which belongs to the dry season, there was no precipitation, there are the lowest values of PM concentration, unlike the months of January and February that did have precipitation.On the other hand, our results are more similar to a study carried out in India, where it was shown that the reduced levels of $PM_{2.5}$, caused by lockdown measures, counteracted the increase in pollutants in the face of adverse weather conditions^53^.

In summary, our results indicate a substantial reduction in air pollution in Cusco city at the beginning of the pandemic, with significant decreases of − 59.52% in $PM_{2.5}$ and − 57.43% in $PM_{10}$. This finding aligns with research internationally such as in the United States, where several key urban areas in the northeast of the country experienced reductions in both $PM_{10}$ and $PM_{2.5}$ during the lockdown period. In Los Angeles, there was a 41% decrease in $PM_{2.5}$ and a 57% decrease in $PM_{10}$. Fresno also exhibited reductions, with a 25% decrease in $PM_{2.5}$ and a 54% decrease in $PM_{10}$. Las Vegas reported notable declines, with a 55% reduction in $PM_{10}$ and a 41% reduction in $PM_{2.5}$^54^. In the city of Delhi, India, a significant decrease of 60% in $PM_{10}$ and 39% in $PM_{2.5}$ was observed compared to 2019.

According to Mahato ^55^, these data emphasize the positive impact of the measures implemented in various global cities to mitigate air pollution during the confinement period. In the case of Peru, the restrictions implemented by the government also had a favorable effect on the atmosphere, as observed in Arequipa, where a reduction in the concentration of $PM_{2.5}$ less than 21.0% and $PM_{10}$ less than 21.5% was documented^28^. On the other hand, in Lima, reductions of up to 40% and 58% were recorded for $PM_{10}$, and 31% and 43% for $PM_{2.5}$, according to the study by Rojas et al.^27^. In contrast to our research, these two cities showed less marked reductions.

## Conclusions

The results of the research have quantitatively demonstrated that the strict confinement at the beginning of the SAR-CoV2 pandemic led to a notable reduction in $PM_{2.5}$ and $PM_{10}$ concentrations at a monitoring point in Cusco city. PM concentration was considerably reduced at the beginning of the pandemic compared to the months before the pandemic, an average percentage decrease of − 59.52% and − 57.43% was obtained for $PM_{2.5}$ and $PM_{10}$ respectively. In dry pre-pandemic periods and the beginning of the pandemic (2018 and 2020), the reduction in $PM_{2.5}$ was 80.87% and in $PM_{10}$ was 68.01%. In normal situations this variation is not considerable due to the concentration of particulate matter coming from the vehicle fleet and brickyards in Cusco city, it was concluded that meteorological parameters did not influence the reduction of the concentration of particulate matter. The reduction of the vehicle fleet to a minimum percentage of passability in the city of Cusco in the first months of the pandemic (2020) was correlated with the reduction in the concentration of particulate matter. The results demonstrate that the mobility restriction measures in automotive transportation implemented by the Peruvian government during the pandemic had a positive impact on the air quality of Cusco city. The progressive return of vehicular transport in mid-May showed an increase in the concentration values of $PM_{2.5}$ and $PM_{10}$, this increase was consolidated in the months of June and July. The SAR-CoV2 pandemic had as a secondary effect the significant reduction in air pollution due to particulate matter at a UNSAAC sampling point. These findings highlight the importance of implementing policies and measures to reduce emissions of air pollutants, especially related to the motor transport sector to improve air quality and promote better public health.
